# Morphine-sensitive synaptic transmission emerges in embryonic rat microphysiological model of lower afferent nociceptive signaling

**DOI:** 10.1126/sciadv.abj2899

**Published:** 2021-08-27

**Authors:** Kevin J. Pollard, Devon A. Bowser, Wesley A. Anderson, Mostafa Meselhe, Michael J. Moore

**Affiliations:** 1 Department of Biomedical Engineering, Tulane University, New Orleans, LA 70118, USA.; 2 Bioinnovation Program, Tulane University, New Orleans, LA 70118, USA.; 3 AxoSim Inc., New Orleans, LA 70112, USA.; 4 Tulane Brain Institute, Tulane University, New Orleans, LA 70118, USA.

## Abstract

Debilitating chronic pain resulting from genetic predisposition, injury, or acquired neuropathy is becoming increasingly pervasive. Opioid analgesics remain the gold standard for intractable pain, but overprescription of increasingly powerful and addictive opioids has contributed to the current prescription drug abuse epidemic. There is a pressing need to screen experimental compounds more efficiently for analgesic potential that remains unmet by conventional research models. The spinal cord dorsal horn is a common target for analgesic intervention, where peripheral nociceptive signals are relayed to the central nervous system through synaptic transmission. Here, we demonstrate that coculturing peripheral and dorsal spinal cord nerve cells in a novel bioengineered microphysiological system facilitates self-directed emergence of native nerve tissue macrostructure and concerted synaptic function. The mechanistically distinct analgesics—morphine, lidocaine, and clonidine—differentially and predictably modulate this microphysiological synaptic transmission. Screening drug candidates for similar microphysiological profiles will efficiently identify therapeutics with analgesic potential.

## Teaser

Bioelectrical signals analogous to pain arise in a bioengineered tissue model of peripheral nerve connected to spinal cord.

## INTRODUCTION

Chronic pain is a debilitating and increasingly prevalent condition that continues to be insufficiently managed with modern analgesics (*1*–*5*). Many leading analgesics have proven highly addictive and/or ineffective for long-term use, and overreliance on powerful and addictive opioid compounds has contributed to an epidemic of prescription drug abuse in the past decade (*6*, *7*). Conventional preclinical drug screening in animal models has failed to meet the urgent need for improved, safe analgesics, and there is increasing recognition of the need for alternative, more efficient preclinical research models (*8*–*12*). Currently, nearly 92% of neurological drugs that reach phase 1 clinical trials fail to reach the market because of unacceptable toxicity or lack of efficacy in humans (*9*). Microphysiological systems (MPS) have the potential to enhance efficiency of preclinical drug screening by combining the physiologically relevant functional data, normally obtained through in vivo experimentation, with the heightened throughput and experimental control of conventional in vitro methodology (*13*–*19*). Future applications of human pluripotent stem cell (hPSC)–derived cell type MPS may even eliminate interspecies differences that confound translational studies and revolutionize patient-specific disease modeling and therapeutic design (*20*).

However, advanced characterization of emergent physiology in rodent MPS is necessary to contextualize the functional data obtained from these systems within the much larger, predominantly rodent model–based, body of basic research on which our understanding of the neurobiology of pain is based. Within the context of MPS, emergent physiology has been described as concerted, macroscopic biological functions that arise through self-directed organization of individual cells into multicellular, cohesive tissues through interactions between cells and with the extracellular environment (*21*). Identification of emergent physiology with immediate clinical relevance will maximize the current potential of MPS-based drug screening, and defining the mechanisms underlying their emergence will inform continued and future development of hPSC-derived MPS.

In vivo, pain processing requires coordinated communication between the peripheral and central nervous systems (PNS and CNS). Pain signals originate in dorsal root ganglion (DRG)–derived nerve fibers of the PNS. Neuronal soma in the DRG projects a single pseudounipolar axon, with one branch innervating peripheral tissue and the other innervating the spinal cord. Axon extensions in peripheral tissue detect noxious stimuli and encode that information as a bioelectric pain signal, which propagates along the axon through the DRG toward the spinal cord and is relayed to the CNS through synaptic transmission in the spinal cord dorsal horn (SCDH). Peripheral pain fibers mainly innervate interneurons of the most superficial layers of the SCDH, and their input is largely glutamatergic and excitatory (*22*). The superficial SCDH interneuron population is a mixture of excitatory glutamatergic and inhibitory GABAergic neurons that form a complex, interconnected, polysynaptic circuit that may gate or amplify afferent pain signals en route to ascending projection neurons located in laminae I and III to V (*23*). Projection neurons extend axons out of the spinal cord along the spinothalamic tract to relay the output of the SCDH circuitry to higher-level brain regions to process the location and emotional aspects of pain (*3*, *24*).

The dorsal spinal cord is a frequent target of pain research, and therapeutic intervention as ascending pain signals may be endogenously gated in the DRG-SCDH circuitry through both descending synaptic input from the brain and local release of neuromodulatory peptides (*25*–*27*). Opioid receptors are highly expressed in the most superficial laminae I and II of the SCDH, and intrathecal administration of exogenous opioids, or stimulation of endogenous opioid release within the SCDH, produces powerful analgesia (*28*–*30*). Opioids synergistically induce descending pain relief through disinhibition of spinally projecting, antinociceptive neurons of the midbrain periaqueductal gray and rostral ventromedial medulla (*31*, *32*) as well as disinhibition of dopaminergic reward pathways of the ventral tegmental area, elevating mood and inducing euphoria (*33*). Unfortunately, the unintended rewarding side effects of systemic opioid administration often lead to repetitive use, abuse, tolerance, and addiction. Development of safer opioid compounds that maximize the analgesic effects of opioids in the spinal cord but minimize the rewarding side effects of opioids in the brain would be invaluable for management of intractable pain.

Here, we validate an advanced microphysiological model of the DRG-SCDH synaptic circuitry demonstrating unidirectional, concerted synaptic communication transmitted long distance between independent neurospheroid populations connected only by directed axonal nerve fiber growth. This is distinct from state-of-the-art, fused organoid (“assembloid”) cultures in both the scale of tissue organization and degree of concerted, discrete, population-level physiological function (*34*, *35*). Furthermore, microphysiological transmission was differentially altered by common analgesics, consistent with their distinct mechanisms of action. This rodent DRG-SCDH microphysiological coculture has immediate potential for physiological screening of experimental analgesics and defines key emergent behaviors critical for understanding future hPSC-derived microphysiological models of lower afferent pain signaling.

## RESULTS

### Fabrication of DRG-SCDH microphysiological cocultures

**Fig. 1:**
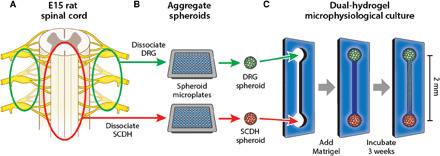
Fabrication of embryonic rat DRG-SCDH microphysiological cocultures. (**A**) All DRG (green) and SCDH (red) nerve tissues are harvested from an entire litter of embryonic day 15 (E15) rat embryos. (**B**) Tissue is pooled by type, dissociated into a single-cell suspension, and aggregated in spheroid microplates to generate a batch of spheroids identical in size and composition. (**C**) A growth-restrictive outer-gel polyethylene glycol mold is fabricated to shape the cultures; spheroids are seeded in the mold, and the mold is filled with growth-permissive Matrigel. Over 3 weeks of culture, microphysiological tissue emerges from which system-level functional data are obtained.

Our laboratory has previously developed embryonic rat microphysiological models of sensory nerve and validated their use for both in vitro neurotoxicity screening and in-depth comparative analysis of chemotherapy-induced peripheral neuropathy (*36*–*40*). Here, we advanced this model by coculturing two distinct neurospheroids, one from DRG and one from SCDH tissue, on either end of the culture system, optimized culture conditions to support three-dimensional axonal growth of both neuronal types, and thoroughly characterized the resulting microphysiological interactions. DRG and SCDH tissues were harvested from an entire litter of gestation day 15 rat embryos. Tissue of each type was separately pooled, dissociated, and reaggregated into a large homogenous batch of equivalent spheroids (Fig. 1). DRG and SCDH spheroids were seeded into opposite ends of a growth-restrictive polyethylene glycol hydrogel mold, which was then filled with a growth-permissive Matrigel-based hydrogel to provide a three-dimensional extracellular matrix (ECM) for neurite migration. Over the course of 3 weeks, spheroids extended neurites and matured into a macroscopic, three-dimensional, anisotropic, microphysiological tissue supporting long-distance nerve conduction and organized interaction between distinct nerve tissue types.

### Emergent recapitulation of DRG-SCDH unidirectional circuit structure and tissue morphology

We first set out to identify ECM conditions that promote emergence of the in vivo structure and function of DRG and SCDH tissues. Spheroids were cultured in several different gelatin methacrylate and Matrigel-based grown permissive hydrogel formulations of increasing stiffness (fig. S1, A to C). Spheroids remained comparably viable in all ECM formulations (fig. S1D), indicating that any physiological differences among conditions are not a result of declining cell health. Consistent with previous reports (*41*–*46*), immunofluorescent staining of total nerve growth with the neuron-specific cytoskeletal protein, β-III-tubulin, confirmed that stiff, gelatin methacrylate–based ECM promoted DRG neurite outgrowth and inhibited SCDH neurite outgrowth in monoculture (fig. S1E). We further confirmed that DRG-SCDH coculture in stiff ECM produced the desired unidirectional neurite growth from DRG to SCDH (fig. S1F), but unexpectedly, bioelectric activity was undetectable in the SCDH spheroid region following electrical stimulation of DRG tissue, which makes evaluation of synaptic physiology impossible. In contrast, soft Matrigel-based ECM permitted robust outgrowth of both DRG and SCDH neurites in monoculture (fig. S1E) and robust detection of bioelectric SCDH field potentials in DRG-SCDH cocultures. Thus, stiff gelatin methacrylate ECM produced the desired unidirectional growth but not the desired physiological behavior, while soft Matrigel ECM produced the desired physiological behavior but did not appear to produce the desired unidirectional growth pattern. These results illustrate how unexpected emergent microphysiological behaviors arise from interaction between both the cells in culture and the ECM composition and that these interactions are critical to accurate interpretation of data produced from MPS.

**Fig. 2:**
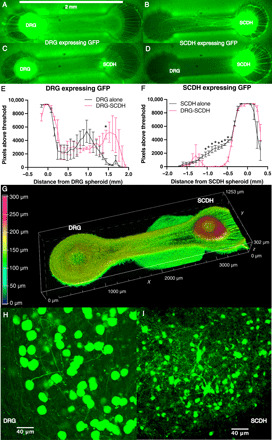
Live tissue imaging of virally expressed GFP in microphysiological DRG and SCDH spheroid cocultures. After 17 days in microphysiological culture, (**A**) DRG and (**B**) SCDH spheroid monocultures extend neurites along the entire length of the hydrogel scaffold. In coculture, (**C**) DRG neurite growth is unaffected by the presence of SCDH spheroids, while (**D**) SCDH neurite outgrowth is inhibited by the presence of the DRG spheroid, resulting in a unidirectional DRG to SCDH neurite growth pattern. In the main growth channel (~0.5 to 1.5 mm), (**E**) there were no significant differences in GFP-expressing DRG neurite density between DRG monocultures and DRG-SCDH cocultures, while (**F**) GFP-expressing SCDH neurite density was significantly decreased in DRG-SCDH cocultures relative to SCDH monocultures. (**G**) A depth-coded three-dimensional rendering of virally encoded GFP expression indicates that the majority DRG tissue (left spheroid) adopts a relatively long, flat morphology less than 150 μm in height while SCDH tissue (right spheroid) maintains a relatively tall, spherical morphology with much of the spheroid near 250 μm in height. (**H**) Neuronal soma in the DRG spheroid region are relatively large and extend a single large axonal process easily distinguished under ×20 magnification. In contrast, (**I**) SCDH neuronal soma is smaller and extends multiple small processes, forming a mesh of dendritic and axonal processes. These results illustrate the emergence of unidirectional DRG-SCDH neurite growth, nerve morphology, and neuronal morphology in microphysiological coculture that is consistent with in vivo DRG and SCDH tissue. *P < 0.05, two-way mixed model analysis of variance (ANOVA) with least significant difference post hoc test.

The apparent potential for bidirectional nerve growth in soft, Matrigel-based ECM could presumably confound the interpretation of field potential recordings in the SCDH spheroid because these potentials may be conflated with direct, electrically evoked activation of SCDH through distal neurite stimulation. Neurite tracing was performed to evaluate the extent of this potentially confounding bidirectional nerve growth. DRG or SCDH spheroids were infected with green fluorescent protein (GFP)–expressing adeno-associated viruses (AAVs) and cultured either alone or in the presence of an uninfected spheroid of the other type. Neurite outgrowth was then tracked over the 3-week maturation period using green fluorescence to identify neurites extending from specified spheroids. Fluorescent neurite outgrowth was quantified after 17 days in microphysiological culture (Fig. 2, A to I). Full statistical analysis of the neurite outgrowth assay, including all relevant *F*, *P*, and df values, is presented in fig. S1.

Compared to DRG monocultures, GFP-expressing DRG tissue in cocultures showed little significant difference in neurite outgrowth except at a single location, approximately 1.5 mm distal from the DRG spheroid (*P* = 0.0107), consistent with the front side of the unlabeled SCDH spheroid (Fig. 2E). In contrast, compared to SCDH monocultures, GFP-expressing SCDH tissue in cocultures showed a consistently significant decrease in neurite growth between 0.5 and 1 mm distal from the SCDH spheroid, corresponding to the shared growth channel (Fig. 2F). These experiments demonstrated that although both DRG (Fig. 2A) and SCDH monocultures (Fig. 2B) extend appreciable nerve growth along the length of the growth-permissive channel, SCDH nerve growth was significantly inhibited in DRG-SCDH coculture (Fig. 2, D and F), while DRG nerve growth remained unaffected (Fig. 2, C and E). The spontaneous recapitulation of unidirectional DRG-SCDH nerve growth in microphysiological culture is an unexpected result and demonstrates an important emergent behavior of these tissues, the directed connectivity characteristic of in vivo nerve connections that underlie lower afferent pain signaling.

In subsequent cultures, AAV-GFP infection rate was reduced to about 20% to generate DRG-SCDH cocultures in which individual neuron cytoarchitecture could be resolved. Three-dimensional rendering of a *z* stack through the mature culture demonstrated that DRG spheroids spontaneously adopted a flat tissue morphology (Fig. 2G, left) relative to SCDH spheroids that retained a more spheroidal morphology (Fig. 2G, right). Maximum intensity projections through higher magnification *z* stacks of the DRG spheroid (Fig. 2H) and the SCDH spheroid (Fig. 2I) demonstrated that DRG soma was larger in size, with DRG soma averaging 18.4 μm and SCDH soma averaging 11.4 μm in diameter (Student’s *t* test, *t* = 3.765, *P* = 0.0197, df = 4), and extended a single large axon into the ECM that is easily distinguished at 20×, while SCDH neuronal soma was smaller in size and extended multiple, fine neurites, forming a mesh-like neuritic arbor. Thus, these two tissues retain distinct characteristics of neuronal and tissue morphology in microphysiological culture that are consistent with in vivo DRG and SCDH nerve tissue.

### Asymmetrical expression of synaptic proteins suggests unidirectional DRG-SCDH connectivity

Qualitative immunofluorescent staining confirmed that both DRG and SCDH tissues expressed the neuronal markers, β-III-tubulin, and microtubule-associated protein 2 (MAP2) (fig. S2, C and I). The peripheral nerve–specific marker, peripherin (fig. S2D), was highly expressed in the DRG, but not SCDH, confirming peripheral and central nerve identity, respectively. The synaptic proteins, synapsin I, postsynaptic density protein 95 (PSD95), and vesicular γ-aminobutyric acid (GABA) transporter, were highly expressed in the SCDH (fig. S2, F, G, and J) suggesting the potential formation of functional anterograde DRG-SCDH synapses. However, relatively minimal expression of these synaptic proteins in the DRG spheroid region suggested limited potential for substantial retrograde SCDH-DRG synapse formation.

**Fig. 3:**
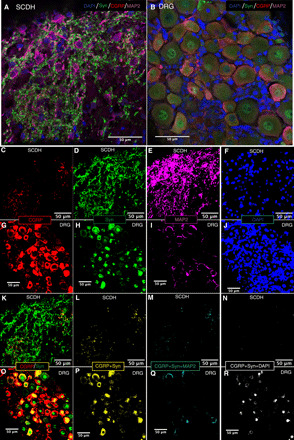
Punctate synaptic markers localize to SCDH but not to DRG spheroids in DRG-SCDH cocultures. Immunofluorescent labeling of the presynaptic marker, synapsin I (Syn); the DRG-derived nerve fiber marker, CGRP; and the dendritic cytoskeletal marker, MAP2, is detectable in both spheroid regions. In composite images, (**A**) highly punctate synapsin I immunoreactivity is detectable on and around MAP2-positive somal and dendritic regions in the SCDH, consistent with synaptic nerve terminals, while (**B**) synapsin I–positive puncta are absent from the DRG spheroid, consistent with a lack of functional synapses. Individual channels comprising the SCDH (**C** to **F**) and DRG (**G** to **J**) composite images were subjected to identical thresholding and converted to binary masks for colocalization analysis. Colocalization of CGRP and synapsin I was observed in both the SCDH spheroid (**K**) and DRG spheroid (**O**) regions. However, CGRP/synapsin I colocalization was punctate in the SCDH (**L**) but expressed diffusely throughout the cell somas of the DRG spheroid (**P**). Furthermore, 39% of CGRP/synapsin I puncta also tricolocalized with MAP2 expression in the SCDH spheroid (**M**), consistent with a postsynaptic connection, while only 2% colocalized with DAPI (**N**), which is more consistent with somal expression. In contrast, only 19% of CGRP/synapsin I puncta colocalize with MAP2 expression in DRG spheroids (**Q**), while 35% colocalize with DAPI (**R**). Together, these data indicate a greater potential for functional synapse formation in the SCDH, including both afferent, synapsin I^+^/CGRP^+^, DRG-SCDH synapses and recurrent, synapsin I^+^/CGRP^−^, SCDH-SCDH synapses, than in the DRG, which preferentially displays intracellular expression of synaptic proteins.

Analysis of colocalization of the presynaptic marker, synapsin I; PNS-specific presynaptic marker, calcitonin gene-related peptide (CGRP); and the dendritic marker, MAP2, was performed to visualize the potential for synaptic connections in DRG-SCDH cocultures. When immunofluorescently stained for these three markers, a highly punctate staining pattern of synapsin I, consistent with synaptic nerve terminals within the MAP2-stained dendritic arbor of SCDH, was observed upon high-magnification imaging of immunofluorescently stained, cryosectioned microphysiological cocultures (Fig. 3A). Although all neurons in the DRG spheroid region were synapsin I positive and a subset of neurons were CGRP positive, there was an absence of punctate staining in the DRG spheroid region, suggesting an absence of synaptic connectivity (Fig. 3B). Colocalization analysis was performed on binarized images (Fig. 3, C to J). Although CGRP staining density was higher in the DRG spheroid (Fig. 3G) than the SCDH spheroid (Fig. 3C) in the presented images, 42% of CGRP expression colocalized with synapsin I expression in SCDH (Fig. 3, K and L), while only 24% of CGRP expression colocalized with synapsin I in the DRG spheroid (Fig. 3, O and P). Furthermore, 39% of CGRP/synapsin I colocalized puncta in the SCDH spheroid also tricolocalized with MAP2 expression (Fig. 3M), indicative of a synaptic connection, while only 3% of CGRP/synapsin I colocalized puncta also tricolocalized with 4′,6-diamidino-2-phenylindole (DAPI), indicative of intracellular expression of these synaptic proteins. In contrast, only 19% of CGRP/synapsin I colocalized pixels also tricolocalized with MAP2 in the DRG spheroid, while a larger proportion, 35%, tricolocalized with DAPI. Together, these data suggest that DRG neurons are producing synaptic proteins and that these proteins are preferentially localized at putative synapses in the SCDH spheroid but preferentially localized at intracellular, nonsynaptic sites in the DRG soma. The colocalization of punctate synaptic markers in the SCDH but not DRG spheroid region indicates the potential for appropriate anterograde DRG-SCDH but not retrograde SCDH-DRG synaptic connections.

### Optogenetics confirm emergence of functional unidirectional DRG-SCDH synaptic neurotransmission

**Fig. 4:**
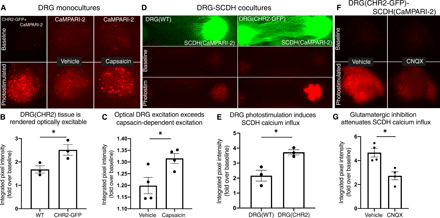
Optogenetic stimulation of DRG tissue excites SCDH nerve tissue through glutamatergic neurotransmission in microphysiological coculture. AAV-dependent expression of a CHR2-GFP fusion protein renders microphysiological nerve tissue optically excitable by 488-nm light, and AAV-dependent expression of CaMPARI-2 renders calcium currents optically recordable through calcium-dependent green-to-red photoconversion during concurrent exposure to 405-nm light. (**A**) Red CaMPARI-2 fluorescence was measured at baseline (top) and after photostimulation (bottom) to quantify CHR2-induced (left), baseline (middle), and capsaicin-induced (right) calcium currents in DRG monocultures. CaMPARI-2–recorded calcium currents are significantly greater than baseline calcium currents during (**B**) activation of CHR2-GFP and (**C**) capsaicin treatment. (B and C) CHR2-induced calcium currents are larger than capsaicin-induced calcium currents confirming that CHR2-induced nerve excitation is large enough for physiological relevance. (**D**) Baseline green (top) and red (middle) fluorescence recorded from coculture of a wild-type DRG spheroid (left) or a DRG spheroid virally expressing CHR2-GFP (right) with a CaMPARI-2–expressing SCDH spheroid. Following photostimulation, a baseline amount of green-to-red CaMPARI-2 photoconversion is detectable in DRG(WT)-SCDH(CaMPARI-2) constructs (bottom left), while a larger amount of CaMPARI-2 photoconversion is detectable in DRG(CHR2-GFP)-SCDH(CaMPARI-2) constructs (bottom right). (**E**) Photostimulation of CHR2-GFP in DRG tissue significantly increases calcium influx in SCDH relative to DRG(WT) controls. (**F**) Baseline and post-photostimulation red fluorescence observed in DRG(CHR2-GFP)-SCDH(CaMPARI-2) after pretreatment with the AMPA/kainate receptor antagonist 6-cyano-7-nitroquinoxaline-2,3-dione (CNQX) (right) or vehicle alone (left). (**G**) Inhibition of glutamatergic neurotransmission with CNQX significantly reduces DRG(CHR2-GFP) photostimulation-induced calcium influx in SCDH. Data are presented as means ± SEM. *P < 0.05 by an unpaired *t* test.

Viral expression of a channelrhodopsin 2–GFP (CHR2-GFP) fusion protein (*47*) renders nerves optically excitable, while viral expression of the calcium integrator, CaMPARI-2 (*48*), renders neuronal calcium currents optically recordable. We first confirmed that viral expression of CHR2-GFP rendered microphysiological DRG tissue optically excitable. After coexpressing both CHR2-GFP and CaMPARI-2 in DRG monocultures, photostimulation-induced calcium influx recorded by CaMPARI-2 was significantly greater in DRG cultures expressing CHR2-GFP than baseline currents in cultures lacking CHR2-GFP (*t* test: *t* = 3.048, *P* = 0.0381, df = 4; Fig. 4, A and B). For comparison, treatment with capsaicin, which is often used as a physiologically relevant stimulus for in vitro DRG neurons (*49*, *50*), also induced significant calcium influx in DRG(CHR2-GFP+CaMPARI-2) monocultures (*t* test: *t* = 2.0851, *P* = 0.0291, df = 6; Fig. 4, A and C). Synchronized CHR2-induced calcium influx was larger than asynchronous capsaicin-induced calcium influx, confirming that optical excitation of DRG tissue is well above the threshold for physiological significance (Fig. 4, A to C).

We then confirmed that concerted optogenetic stimulation of DRG nerve tissue produces a robust physiological response in cocultured SCDH tissue. Six CaMPARI-2–expressing SCDH spheroids were cocultured with either optically excitable DRG tissue [*n* = 3, denoted as DRG(CHR2-GFP)-SCDH(CaMPARI-2) constructs] or wild-type DRG tissue [*n* = 3, denoted as DRG(WT)-SCDH(CaMPARI-2) constructs]. DRG(WT) tissue did not respond to optical excitation, and only baseline levels of calcium influx were recorded in the SCDH spheroid (Fig. 4D). In contrast, optical excitation of DRG tissue in DRG(CHR2-GFP)-SCDH(CaMPARI-2) constructs significantly increased calcium influx in SCDH tissue (*t* test: *t* = 3.881, *P* = 0.0178, df = 4; Fig. 4, D and E). This confirms that optical excitation in DRG tissue induces active calcium influx in cocultured SCDH tissue, consistent with synaptic transmission.

We lastly confirmed that optically excitable DRG tissue activates calcium currents in SCDH tissue through glutamatergic synaptic transmission. Ten additional DRG(CHR2-GFP)-SCDH(CaMPARI-2) cultures were generated. Half of these cocultures were pretreated with the AMPA/kainate receptor antagonist, 6-cyano-7-nitroquinoxaline-2,3-dione (CNQX), to inhibit glutamatergic neurotransmission, while the other half were treated with vehicle alone [0.2% DMSO (dimethyl sulfoxide)] for 15 min before photostimulation. CNQX pretreatment significantly reduced calcium influx in SCDH following optical excitation of DRG tissue relative to treatment with vehicle alone (*t* test, *t* = 3.945, *P* = 0.0076, df = 6; Fig. 4, F and G). This result confirms that photostimulated DRG nerve tissue excites SCDH nerve tissue through glutamatergic neurotransmission in this microphysiological coculture.

### Distinct and replicable local field potentials recorded in cocultures reflect the local underlying physiology

**Fig. 5:**
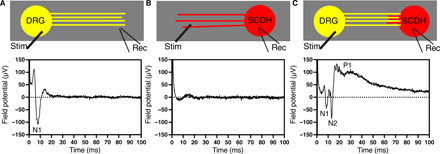
A distinct, replicable, three-part waveform emerges in the SCDH spheroid region of DRG-SCDH cocultures. (**A**) Only a single, fast, negative-going field potential, denoted as N1, is recorded in the nerve region of DRG monocultures upon stimulation of the DRG spheroid region. (**B**) Concerted field potentials were not recorded in the spheroid region of SCDH monocultures upon stimulation of the nerve region. When DRG and SCDH spheroids are cocultured, (**C**) a three-part waveform is recorded in the SCDH spheroid region upon stimulation of the DRG spheroid region. This waveform is composed of two fast, negative-going field potentials, denoted as N1 and N2, and a slower, prolonged, positive-going field potential, denoted as P1. N1 is likely a recording of the concerted depolarization of DRG nerve fibers running through the SCDH spheroid region that are observed in the absence of the SCDH spheroid, as shown in (A). N2 and P1 are not observed in the absence of the DRG spheroid and are therefore potentially a result of microphysiological synaptic transmission between DRG and SCDH nerve tissue.

Extracellular field potential production was qualitatively evaluated throughout DRG and SCDH monocultures, DRG-DRG cocultures, SCDH-SCDH cocultures, and DRG-SCDH cocultures to identify a potential synaptic waveform specific to the DRG-SCDH configuration. Electrically evoked compound action potentials (CAPs) were readily recorded in DRG spheroid monocultures. CAP conduction was detected both in the DRG spheroid following stimulation of the nerve region (fig. S3A) and in the nerve region following stimulation of the DRG spheroid in mature DRG monocultures (Fig. 5A). Field potentials recorded in the spheroid region consisted of a large negative-going population spike followed by several smaller negative-going peaks, while field potentials recorded in the nerve outgrowth consisted of a single similar large negative-going population spike lacking subsequent smaller peaks. Consistent with electrically evoked action potentials, these CAPs are insensitive to EDTA and CNQX but can be completely blocked by tetrodotoxin (fig. S4).

Electrically evoked CAP conduction was not detectable in the nerve (fig. S3D) or spheroid region (Fig. 5B) of SCDH spheroid monocultures after direct stimulation despite the observation that nerve growth extends the entire length of the growth-permissive channel (Fig. 2B). It is likely that the SCDH nerve tissue was activated by the electrical stimulation, but the resulting field potentials were not large or synchronous enough to be detected through extracellular recording.

Waveforms recorded in the DRG spheroid region of DRG-SCDH cocultures (fig. S3H, recording A) were nearly identical to waveforms recorded in DRG monocultures (fig. S3A), indicating that the DRG field potential was relatively unaffected by coculture with SCDH tissue. However, a consistent and unique waveform indicative of synaptic transmission emerged in SCDH spheroids after coculture with DRG tissue (Fig. 5C). This waveform was recorded in the SCDH after stimulation of DRG spheroids, was replicable, and was distinct from waveforms observed in DRG or SCDH monocultures, DRG-DRG cocultures, or SCDH-SCDH cocultures (fig. S3, A to F). This waveform consisted of two, fast negative-going population spikes (denoted as N1 and N2) followed by a slower but prolonged positive-going wave (denoted as P1). Stimulation of the distal DRG neurite growth in fig. S3I confirms that this synaptic waveform was not conflated with electrically evoked SCDH spheroid activation resulting from direct stimulation of distal SCDH neurite growth. This distal stimulation site was well beyond the range of SCDH neurite outgrowth observed in DRG-SCDH coculture (Fig. 2D) but still produced a SCDH spheroid waveform containing the same three components as more proximal stimulation sites, albeit with the longer latency and reduced amplitude expected from a more distal stimulation. The 2-mm stimulation site was found to be optimal, as it produced the most synchronous waveform that permitted the best resolution of the individual components and was therefore used for all subsequent experiments (fig. S3I).

### Fatigue associated with repeated stimulation further distinguishes synaptic waveform components

**Fig. 6:**
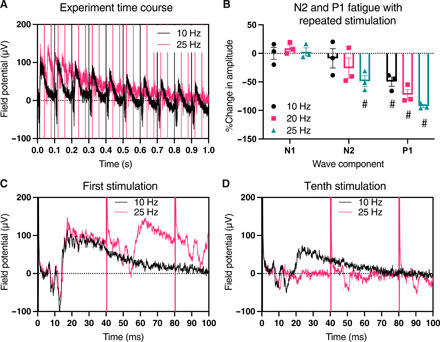
Synaptic waveform components N2 and P1, but not electrically evoked N1, fatigue under repeated stimulation. DRG spheroids were repeatedly stimulated for 1 s at increasing frequencies of 10, 20, and 25 Hz while continuously recording field potential production in the SCDH of DRG-SCDH cocultures. (**A**) Comparison of the overall time course of the 10 Hz (black) and 25 Hz (red) experiments for a representative construct. (**B**) The amplitude of the N1, N2, and P1 waveform components was measured after each stimulation and normalized to initial amplitudes to calculate the percent change in each component relative to baseline. N1 amplitude remains unchanged across the first 10 stimulations at each stimulation frequency. A significant reduction in N2 amplitude is observed after the 10th stimulation at 25 Hz, and a significant reduction in P1 amplitude is observed after the 10th stimulation at frequencies of 10 Hz and above. (**C**) Baseline waveforms recorded after the first stimulation at 10 Hz (black) and 25 Hz (red) are similar to clearly identifiable N1, N2, and P1 peaks. (**D**) N2 and P1 are absent after the 10th stimulation at 25 Hz (red), and P1 is still present but significantly reduced after the 10th stimulation at 10 Hz (black). Data are presented as means ± SEM. #*P* < 0.05, one-sample *t* test versus 0%.

The synaptic nature of the unique DRG-SCDH waveform was first challenged with high-frequency stimulation, which is expected to fatigue synaptically based components more readily than electrically evoked components (*51*, *52*). The DRG spheroid region of DRG-SCDH coculture constructs was sequentially stimulated for 1 s at increasing frequencies of 10, 20, and 25 Hz (Fig. 6A). The amplitude of N1, N2, and P1 was measured for each stimulation in the train, and amplitudes were normalized to initial amplitude to obtain a percent change in amplitude across the stimulation train (Fig. 6B). N1 amplitude remained unchanged across the first 10 stimulations at all frequencies (Fig. 6, B to D). In contrast, a significant reduction in N2 was observed at 25 Hz (one-sample *t* test versus 0%, *t* = 5.031, *P* = 0.0373, df = 2), and a significant reduction in P1 amplitude was observed at frequencies of 10 Hz and above (10 Hz: *t* = 5.973 *P* = 0.0373, df = 2; 20 Hz: *t* = 8.233 *P* = 0.0144, df = 2; 25 Hz: *t* = 33.14, *P* = 0.0009, df = 2). These results suggest that N1 is a result of electrically evoked CAP conduction, while N2 and P1 result from synaptic transmission.

### Selective pharmacological inhibition dissociates synaptic waveform components from fiber volley

**Fig. 7:**
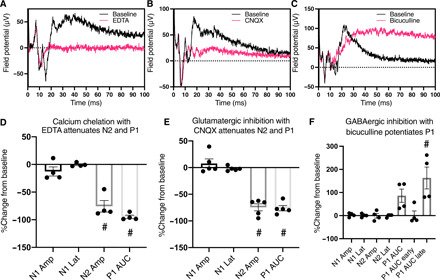
Both glutamatergic and GABAergic neurotransmission shape the DRG-SCDH microphysiological synaptic waveform. DRG nerve tissue was electrically stimulated and resulting field potentials were recorded in the SCDH of DRG-SCDH cocultures in the presence of (**A**) the calcium chelator, EDTA, (**B**) the AMPA/kainate receptor antagonist, CNQX, or (**C**) the GABA_A_R antagonist, bicuculline. Inhibition of all synaptic transmission with EDTA (**D**) or inhibition of glutamatergic AMPA/kainate receptor signaling with CNQX (**E**) significantly reduces the amplitude of the N2 and P1 peaks while having no effect on N1. (**F**) Inhibition of GABA_A_R signaling with bicuculline specifically enhances the late phase of the P1 peak while having no effect on N2 or N1. Amp, amplitude; Lat, latency; AUC, area under the curve. Data are presented as means ± SEM. #*P* < 0.05, one-sample *t* test versus 0%.

The synaptic basis of the unique DRG-SCDH waveform was further challenged through selective pharmacological inhibition of synaptic transmission. Bath application of EDTA chelates free calcium, preventing calcium-dependent release of synaptic vesicles. The AMPA/kainate receptor antagonist, CNQX, was applied to specifically block glutamatergic neurotransmission, while the GABA receptor type A (GABA_A_R) antagonist, bicuculline, was applied to specifically block GABAergic neurotransmission. Blockade of synaptic transmission with EDTA (Fig. 7, A and D) completely blocked the N2 population spike (one-sample *t* test versus 0%, *t* = 7.309, *P* = 0.0053, df = 3) and the P1 wave (*t* = 30.53, *P* < 0.0001, df = 3). Similarly, blockade of glutamatergic neurotransmission with CNQX (Fig. 7, B and E) significantly reduced N2 (*t* = 11.23, *P* = 0.0004, df = 3) and P1 (*t* = 15.42, *P* = 0.0001, df = 4). In contrast, blockade of GABAergic neurotransmission with bicuculline (Fig. 7, C and F) specifically and significantly increased the late phase of the P1 wave (*t* = 3.433, *P* = 0.0415) while having no effect on N1 or N2 (Fig. 7, C and F). None of these treatments affected the latency or amplitude of N1.

N1 was insensitive to high-frequency stimulation (Fig. 6) and all modulators of synaptic activity (Fig. 7) but was completely blocked by the voltage-gated sodium channel inhibitor, tetrodotoxin (fig. S4). This is consistent with direct recording of the electrically evoked, concerted depolarization of presynaptic afferent DRG nerve fibers growing through the SCDH spheroid region or fiber volley. In contrast, N2 and P1 fatigue under repeated stimulation and are not observed when synaptic vesicle release is prevented by calcium chelation, confirming that they are synaptic in nature. N2 is negative going, similar in size and time locked to N1, inhibited by glutamatergic inhibition, but insensitive to GABAergic inhibition. This is consistent with a concerted primary field excitatory postsynaptic potential (fEPSP) resulting from glutamatergic neurotransmission from DRG to SCDH tissue. P1 is synaptically evoked but is delayed and prolonged relative to N1 and N2, continuing tens of milliseconds after termination of N2. This likely represents a recording of higher-order, multisynaptic impulses propagating through the SCDH, which is prolonged by disinhibition after blockade of inhibitory neurotransmission with bicuculline. Together, these results strongly suggest that functional and organized synaptic physiology is an emergent behavior of DRG and SCDH tissue in microphysiological coculture.

### Common analgesics induce overlapping but distinct effects on synaptic physiology

Preliminary qualitative dose-response experiments were performed to identify the lowest effective dose of each analgesic and to demonstrate the reversibility of drug effects with at least a partial washout (figs. S5 and S6). The lowest effective dose was then replicated in four separate microphysiological cultures. A full stimulus-response curve was performed for each sample at baseline, after a sham treatment, and after analgesic treatment, and the change in latency and amplitude of N1, N2, and P1 wave components as well as the integrated area under the curve (AUC) of P1 were calculated for each stimulus intensity at each stage of treatment.

**Fig. 8:**
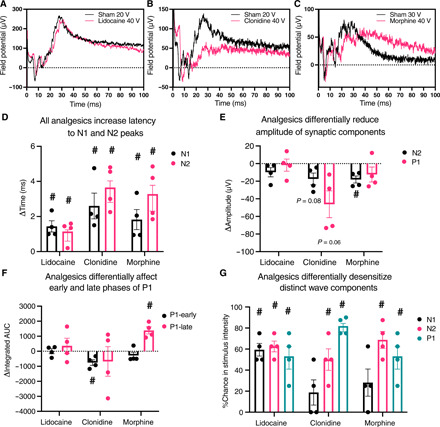
The commonly used analgesics lidocaine, clonidine, and morphine differentially modulate synaptic transmission in a microphysiological model of lower afferent pain signaling. Differential desensitization of the afferent DRG input was controlled for by matching the maximal posttreatment to its pretreatment trace with the most similar N1 amplitude. Example N1-matched lidocaine (**A**), clonidine (**B**), and morphine (**C**) traces. Statistical analysis of N1-matched traces indicated that (**D**) all analgesics significantly increased the latency to both N1 and N2 peaks, (**E**) clonidine more specifically reduced the maximum amplitude of P1 while morphine significantly reduced the amplitude of N2, and (**F**) clonidine significantly attenuated the early phase of P1 while morphine significantly increased the late phase of P1. N1, N2, and P1 amplitude matching was performed, and the difference in stimulus intensity required to produce an N1, N2, and P1 peak of equal amplitude before and after analgesic treatment was calculated to quantify differential desensitization of each of the peak components. (**G**) Statistical analysis of the change in required stimulus intensity indicated that lidocaine primarily desensitized N1 while clonidine and morphine primarily desensitize N2 and P1. Data are presented as means ± SEM. #*P* < 0.05 one-sample *t* test versus 0 (no change).

Quantitative analysis of the stimulus-response curves revealed evidence of analgesic-induced attenuation of the fiber volley peak across stimulus intensities, suggesting that interpretation of the postsynaptic effect of analgesics is partially conflated with presynaptic desensitization (fig. S7). Therefore, the maximum response observed after analgesic treatment was paired with the sham stimulation intensity that had previously produced an N1 fiber volley peak of the most similar amplitude, and differences in the synaptic components of the waveform were compared between N1-matched traces to better isolate changes to the postsynaptic response to an input of similar intensity (Fig. 8, A to C). Significant changes in waveform component metrics between N1-matched sham and analgesic traces were identified with a one-sample *t* test versus 0 (indicating no change) composed of *n* = 4 paired traces for each drug treatment (df = 3), and all exact *P* values resulting from this analysis are shown in table S2. Statistical analysis confirmed that all three analgesics significantly increased the latency to N1 and N2 (Fig. 8D). Lidocaine had no additional significant effects on the shape of the synaptic waveform. In contrast, additional changes were evident after treatment with both clonidine and morphine. Clonidine preferentially decreased P1 amplitude, while morphine significantly reduced N2 amplitude (Fig. 8E), and clonidine uniquely decreased the early phase of P1, while morphine uniquely increased the late phase of P1 (Fig. 8F). These results indicated that lidocaine acts primarily on presynaptic DRG tissue, while clonidine and morphine affect the postsynaptic response in different ways independent of changes in presynaptic input strength.

Last, the maximal posttreatment amplitude of each waveform component was independently matched with the associated pretreatment trace that evoked a response of the most similar magnitude. The percent change in stimulus intensity required to evoke this response of similar amplitude was then calculated to independently quantify changes in the sensitivity of each distinct waveform component. Lidocaine uniquely decreased the sensitivity of the presynaptic fiber volley, requiring approximately 60% greater stimulus intensity to observe an N1 peak of similar magnitude (Fig. 8G). A similar desensitization of N2 and P1 was observed, indicating that the desensitization of N1 drives the desensitization of the dependent N2 and P1 peaks. In contrast, N2 and P1 were primarily desensitized after treatment with clonidine and morphine, while no significant effect on N1 sensitivity was observed. These results confirm that lidocaine primarily desensitizes presynaptic DRG nerve tissue, while clonidine and morphine directly desensitize the postsynaptic response.

## DISCUSSION

Here, we present several converging lines of evidence indicating that emergent properties of microphysiological DRG-SCDH spheroid cocultures self-assemble an efficient and informative model of lower afferent pain signaling in our defined MPS. Microphysiological cocultures spontaneously assemble into a unidirectional circuit with extensive anterograde (DRG-to-SCDH) but limited retrograde (SCDH-to-DRG) neurite extension (Fig. 2, A to F). DRG and SCDH spheroid tissues retain distinct morphologies that are consistent with in vivo DRG and SCDH tissue structure and cytoarchitecture (Fig. 2, G to I). Colocalization of pre- and postsynaptic markers in the SCDH but not DRG spheroid region confirms the potential for anterograde but not retrograde synapse formation (Fig. 3). A specific and unique synaptic field potential composed of at least two, fast negative-going peaks (N1 and N2) followed by a prolonged positive-going wave (P1) is generated in the SCDH spheroid region after stimulation of cocultured DRG tissue (fig. S3). Functional and pharmacological challenges confirmed the N1 peak to be presynaptic fiber volley and the N2 peak to be a primary glutamatergic fEPSP, while the less synchronous P1 peak likely represents secondary and higher-order recurrent synaptic activity within the SCDH spheroid (Figs. 5 to 7). Last, proof-of-concept, small-scale analgesic screening identified overlapping but distinct effects on microphysiological synaptic transmission induced by lidocaine, clonidine, and morphine that are consistent with in vivo descriptions of their distinct mechanisms of actions (Fig. 8).

In vivo, nociceptive signals originate in peripheral pain fibers and are transmitted unidirectionally to the CNS via synaptic transmission in the SCDH (*53*). Consistent with a single axis of meaningful information transfer, each large DRG soma extends a single large axon, and axons of many cells converge into relatively flat nerve tissue ultrastructure, reaching a vertical depth of only 50 μm despite several millimeters of horizontal growth (Fig. 2, G and H). In contrast, the in vivo SCDH is a multilaminate structure in which information transfer occurs three-dimensionally, both collaterally to neurons within the same layer and vertically to neurons of deeper and more superficial layers (*54*). Consistent with multiple axes of information transfer, small SCDH soma extends multiple, fine, ramified neuronal fibers that coalesce into a three-dimensional neurite meshwork, reaching ~300 μm in height despite very limited horizontal outgrowth (Fig. 2, G and I). Emergent cellular and tissue morphology forms a tissue structure that permits both unidirectional information transfer between DRG and SCDH tissues and multidirectional information transfer within microphysiological SCDH tissue consistent with in vivo descriptions of their structure-function relationship.

This organized tissue ultrastructure facilitates emergence of the most valuable feature of MPS: in vitro reproduction of organized, complex, tissue-level physiology that is not possible with conventional culture methodology (*55*). Neurophysiology can be characterized, to an extent, using conventional dispersion culture combined with microelectrode array or intracellular electrophysiological recording techniques. However, the physiology of a single neuron cultured in relative isolation on two-dimensional substrates cannot approximate the complexity of, and is often much different from, the behavior of the same cell population within the context of a fully developed tissue (*56*–*59*). Here, we demonstrate organized tissue-level physiological interaction between DRG and SCDH cell populations in our defined MPS. This interaction occurs between distinct neurospheroid types separated by two millimeters and connected only by directed nerve fiber growth. This level of discrete tissue organization and connectivity exceeds that of assembloid MPS that consist of fused organoids grown in direct contact with each other or differentiated into distinct cell types in different parts of the same spheroid from the same pool of progenitors (*34*, *35*). Critically, through both optogenetic and electrophysiological methods, we confirmed that concerted stimulation of DRG tissue results in a discrete, concerted physiological response in cocultured SCDH tissue through glutamatergic neurotransmission.

Optogenetic calcium imaging and field potential recording are complimentary approaches that together enable direct, rapid, highly informative, and clinically relevant physiological readouts appropriate for drug screening or preclinical research. The optogenetic approach affords ultimate control over tissue type–specific stimulation and physiological response recording, applied here to provide robust confirmation of the directionality of synaptic neurotransmission. Specific photostimulation of DRG tissue through viral CHR2-GFP expression robustly activated calcium influx in SCDH, specifically recorded through viral expression of CaMPARI-2, that was blocked after inhibition of glutamatergic neurotransmission. The optical clarity and ease of incorporating a variety of genetically modified neurons into this MPS afford extensive future opportunity for advanced optogenetic characterization of DRG-SCDH circuit physiology. With appropriately designed optogenetic tools, the physiology of individual neurons or neuronal subtypes can be directly evaluated within the context of overall circuit function, enabling detailed mechanistic description of analgesic actions in microphysiological DRG-SCDH circuits.

Extracellular field potential recording can be performed with higher throughput, enabling rapid identification of potential novel analgesics, and provides the greater time resolution necessary to differentiate the contribution of distinct underlying biological processes. We determined that glutamatergic input from the DRG tissue first generates an initial primary fEPSP in the SCDH spheroid, which then initiates secondary and higher-order recurrent synaptic transmission, which is limited in duration by GABAergic signaling. Dorsal horn field potentials recorded in ex vivo slice preparations from the rat spinal cord vary substantially on the basis of recording lamina and stimulation site (*60*), and this MPS does not currently replicate the complexity of a fully developed rat spinal cord. However, both ex vivo rat dorsal horn and the microphysiological synaptic waveform are composed of fast synaptic components followed by a longer, slower, positive deflection that are not observed after blockade of calcium influx or glutamatergic inhibition and modulated by GABA_A_R inhibition (*61*). We hypothesized that these common electrophysiological features shared by microphysiological DRG-SCDH cocultures and ex vivo rat spinal cord slices can be exploited to infer analgesic potential of therapeutics.

We tested this hypothesis by characterizing the effect of known analgesics (lidocaine, clonidine, and morphine) on synaptic transmission in this MPS. The analgesic properties of the local anesthetic, lidocaine, are primarily mediated by impairment of action potential propagation through peripheral pain fibers by enhancing the hyperpolarizing after potential but is not thought to directly affect synaptic transmission (*62*). Similarly, in microphysiological DRG-SCDH coculture, lidocaine primarily reduced the sensitivity of the presynaptic fiber volley peak, N1 (Fig. 8G and fig. S7). After controlling for the magnitude of N1, there were no direct effects of lidocaine on the synaptic components of the microphysiological waveform identified (Fig. 8, D to F). The α_2_ adrenergic receptor agonist, clonidine, not only impairs action potential propagation through peripheral nerve fibers but also inhibits voltage-gated calcium channels, impairing presynaptic neurotransmitter release in addition to desensitization of afferent pain fibers (*62*–*64*). The synaptic effects of clonidine predominate in microphysiological DRG-SCDH coculture. Synaptic components N2 and P1 were primarily desensitized after clonidine treatment (Fig. 8G). After controlling for N1 amplitude, there was a trending decrease in the amplitude of the primary fEPSP, N2, and the higher-order recurrent synaptic transmission peak, P1, and a significant decrease in the early (but not late) phase of P1 (Fig. 8, E and F). Thus, this MPS readily distinguished the electrophysiological profile of a local anesthetic that primarily targets desensitization of afferent pain fibers from an α_2_ adrenergic receptor agonist that primarily affects synaptic transmission.

In vivo, opioid receptors are found both presynaptically on DRG-derived afferent nerve terminals and postsynaptically on resident SCDH neurons (*65*). Activation of presynaptic opioid receptors has been shown to impair afferent DRG-derived mechanoreceptor synaptic input to the SCDH through inhibition of voltage-gated calcium channels (*66*) but is not thought to impair CAP propagation along the length of DRG-derived nociceptors. Opioid receptor–dependent inhibition of GABAergic interneurons is a well-established mechanism of opioid-induced disinhibition of excitatory neurons in both the brain and the spinal cord (*31*, *33*). Morphine perfusion in microphysiological DRG-SCDH primarily desensitized the synaptic components N2 and P1 of the microphysiological waveform, similar to clonidine (Fig. 8G). After controlling for N1 amplitude, morphine significantly reduced the primary fEPSP and significantly reshaped the P1 peak by specifically increasing the late (but not early) phase (Fig. 8, E and F). Impairment in primary fEPSP production is consistent with reduced presynaptic release of neurotransmitter, while prolonged propagation of higher-order synaptic activity is consistent with disinhibition of SCDH circuits. The enhanced late phase of P1 strongly resembled the effect of direct GABA_A_R inhibition with bicuculline (Fig. 7, C and F). Thus, it is likely that opioid receptor activation disinhibits microphysiological SCDH through inhibition of GABAergic neurotransmission similar to in vivo descriptions of opioid effects on the brain and spinal cord (*31*, *33*). We hypothesize that screening potential novel analgesics for similar effects on synaptic transmission in this MPS will identify therapeutics with greater analgesic potential before exhaustive testing in animal models, thereby increasing the efficiency of analgesic development.

The microphysiological approach offers distinct advantages over conventional in vitro and in vivo model systems that may augment and streamline large-scale analgesic screening. While necessary to validate the physiological relevance of emerging analgesics and evaluate off-target effects, in vivo experimentation cannot be conducted with high enough throughput to evaluate all candidate compounds. Conversely, conventional two-dimensional in vitro experimentation can be conducted at large scale but is unable to recapitulate tissue-level nerve function and may not accurately represent in vivo cellular neurophysiology (*67*–*70*). Three-dimensional in vitro model systems more closely approximate in vivo physiological function and may therefore more accurately identify compounds with analgesic potential. The results described here represent the first descriptions of analgesic-responsive, concerted, tissue-level DRG-SCDH synaptic transmission measured in vitro in a manner that is scalable for high-throughput experimentation. As many as 50 identical microphysiological cultures can be generated from a single litter of embryonic rats, each of which can be physiologically analyzed in a matter of minutes.

The application of MPS has exciting potential for basic research into the neurobiology of pain and efficient screening of novel analgesics. The use of hPSC-derived tissue in MPS has recently garnered considerable interest, as it stands to eliminate interspecies differences that often confound translational research and enable patient-specific disease modeling and therapeutic intervention (*20*, *71*). However, nearly everything we know about the neurobiology of pain has been described using preclinical animal models. Information obtained from hPSC-derived MPS cannot be properly contextualized within the literature without a deep understanding of emergent behavior of both hPSC and animal-derived cells in MPS (*21*). Here, we show how critical emergent behavior of embryonic rodent DRG and SCDH tissue in MPS enables tissue to spontaneously assemble into a neuronal circuit that recapitulates the critical aspects of in vivo DRG-SCDH circuit physiology necessary to profile the effects of distinct analgesics. Animal-derived cellular models such as these not only can be used immediately for drug screening or basic research but also inform the design of analogous human MPS and provide a bridge of understanding to inform how arising human MPS relate to the vast body of research performed using animal models on which our basic understanding of neurophysiology is based.

## MATERIALS AND METHODS

### Experimental design

This study aimed to develop an in vitro microphysiological model of tissue-level synaptic transmission between peripheral nociceptors and dorsal spinal cord neurons and validate potential future use in analgesic screens. We first sought to identify an optimal ECM composition that promotes the emergence of the appropriate structure-function relationship between DRG and SCDH nerve tissue. Our laboratory has previously had success growing three-dimensional nerve cultures in both gelatin methacrylate and Matrigel-based hydrogels and hypothesized on the basis of previous reports (*41*–*46*) that modulation of gel stiffness could be used to direct neurite growth. Therefore, we designed gelatin methacrylate and Matrigel-based gel formulations that would result in ECM of various stiffness and compared morphology and physiology of cultures across gel formulations to identify the optimal ECM. We next sought to confirm that cultures adopted the robust, unidirectional DRG-SCDH tissue growth in the identified optimal ECM. Unidirectionality of nerve connectivity and information transfer is characteristic of in vivo DRG-SCDH circuits, and we hypothesized that this tissue structure would also give rise to the desired unidirectional synaptic connectivity in microphysiological culture. To distinguish neurites emanating from different spheroids in the shared growth channel, select spheroids were infecting with GFP-expressing AAV before seeding in microphysiological culture. We then compared the relative extension of DRG-derived and SCDH-derived neurites into the shared growth channel and evaluated any evident cellular and tissue-level morphological differences. We next aimed to confirm that concerted, afferent DRG-SCDH, tissue-level synaptic function was present in microphysiological cultures under optimal ECM conditions. Multiple orthogonal optogenetic, electrophysiological, and pharmacological approaches were applied to selectively excite the DRG neuronal population and selectively inhibit neurotransmission to confirm that DRG excitation results in SCDH excitation through synaptic communication. Last, we reasoned that any potential model of lower afferent nociceptive signaling would need to be responsible to common analgesics and hypothesized that common analgesics with distinct underlying mechanisms of action would result in differential modulation of synaptic physiology in this microphysiological coculture. We identified lidocaine, clonidine, and morphine as three common analgesics with distinct underlying mechanisms. We applied these analgesics to our microphysiological cultures and quantified and compared consistent changes to the sensitivity and shape of the characteristic microphysiological synaptic waveform.

### Microphysiological coculture system

All experiments were performed using a three-dimensional dual-hydrogel MPS (Fig. 1). Fabrication methods have previously been extensively described and validated (*36*–*40*, *72*, *73*). Briefly, all DRG and SCDH nerve tissues were harvested from entire litters of embryonic day 15 (E15) rat embryos. All tissues were pooled by type, dissociated into single-cell suspensions, and reaggregated in spheroid microplates generating 30 to 50 spheroids of each type, identical in size and composition. Growth-restrictive outer-gel molds were patterned through digital projection lithography to define the shape of permissive growth area; spheroids were seeded in molds, and molds were filled with growth-permissive Matrigel. Spheroids extended neurites throughout the growth-permissive inner gel, forming microphysiological nerve tissue over the course of a 3-week maturation period.

### Spheroid formation

All animal handling and tissue harvesting procedures were performed according to guidelines set by the U.S. National Institutes of Health (NIH) and approved in advance by the Institutional Animal Care and Use Committee at Tulane University. DRG and SCDH tissues from an E15 rat litter were separately harvested, pooled, and digested in 0.25% trypsin in phosphate-buffered saline (PBS) (pH 7.4) at 37°C for 15 min. After digestion, tissue was pelleted at 500*g* for 5 min; trypsin was removed; and tissue was gently resuspended in growth media, dissociated through trituration, and passed through a 40-μm nylon mesh filter. DRG and SCDH cells were then separately seeded in 96-well ultralow attachment spheroid microplates (Corning Inc., Corning, NY, USA) at a concentration of 45,000 DRG cells per well and 60,000 SCDH cells per well in growth media composed of Neurobasal medium supplemented with 2% v/v B27 supplement, 1% v/v N2 supplement, 1% v/v GlutaMAX, nerve growth factor 2.5*S* native mouse protein (20 ng/ml), recombinant human/murine/rat brain–derived neurotrophic factor (10 ng/ml; PeproTech, Cranbury, NJ, USA), recombinant human glial cell–derived neurotrophic factor (10 ng/ml; PeproTech), and 1% v/v antibiotic/antimycotic solution (all from Thermo Fisher Scientific, Waltham, MA, unless otherwise noted). Microplates were centrifuged at 500*g* for 5 min, and spheroids were aggregated after overnight incubation at 37°C and 5% CO_2_.

### Dual-hydrogel coculture fabrication

Outer gels were fabricated on Costar Transwell clear polyester membrane six-well plate inserts (Thermo Fisher Scientific) by irradiating a solution of 10% w/v polyethylene glycol dimethacrylate (Polysciences Inc., Warrington, PA, USA), 1.1 mM lithium phenyl-2,4,6-trimethylbenzoylphosphinate (LAP; Allevi, Philadelphia, PA, USA), and 0.0001% w/v 2,2,6,6-tetramethylpiperidine 1-oxyl (TEMPO) (MilliporeSigma, St. Louis, MO, USA) in PBS (pH 7.4) with ultraviolet (UV) light patterned by a digital micromirror device (DMD; DLP 4500, Wintech Digital Systems Technology Corp., San Marcos, CA). The growth-restrictive polyethylene glycol gel will only polymerize when exposed to UV light; therefore, the pattern of UV light projected by the DMD dictates the shape of the growth-restrictive mold. The DMD was used to pattern gel molds composed of two bulbous regions, large enough for spheroid placement, connected by a long, thin channel that allows neurite growth between the spheroids (Fig. 1C). The desired spheroids were placed in the bulbs, and the void was filled with a growth-permissive inner-gel solution. The inner-gel was cured. Growth media were applied, and cultures matured for 17 to 21 days in growth medium at 37°C and 5% CO_2_ before morphological or physiological analysis.

During initial growth-permissive gel stiffness optimization, both gelatin methacrylate and Matrigel-based inner gels of varying stiffness were prepared. Matrigel stiffness was modulated by increasing the concentration of Matrigel. Corning Matrigel hESC-Qualified Matrix (Thermo Fisher Scientific) was diluted 1:2, 1:1, and 1:0 in growth media, applied to the inner-gel void, and cured at 37°C and 5% CO_2_ for 15 min before application of growth media. Gelatin methacrylate stiffness was modulated by increasing the concentration of 1-vinyl-2-pyrrolidinone in the gel precursor solution. Gelatin methacrylate (4% w/v; Allevi) and laminin (4 μg/ml; Thermo Fisher Scientific) were dissolved in 1.1 mM LAP and 1-vinyl-2-pyrrolidinone (MilliporeSigma) was added at 0, 0.83, 1.66, or 2.5% v/v. The gel precursor solutions were added to the inner-gel void; gels were cured by irradiation with UV light from the DMD for 30 s, and growth media were immediately added to cured dual-hydrogel cultures. Fifty percent Matrigel was determined to be the optimum ECM formulation and was used as the growth-permissive inner gel for all subsequent morphological and physiological analyses.

### Rheological characterization of gels

The viscoelastic properties of the gelatin methacrylate and Matrigel gel formulations were assessed using a shear rheometer (TA Instruments, AR2000, New Castle, DE). Circular samples (diameter, 4 cm) were prepared within a silicone mold on top of a Parafilm sheet (Bemis Company Inc., Neenah, WI). Gelatin methacrylate samples were exposed to 30 s of UV light (385 nm) to initiate cross-linking. Matrigel was allowed to self-assemble at 37°C for 30 min. For rheological testing, samples were loaded between the plate and the cone (1° steel cone; diameter, 4 cm; room temperature) before running a 0.1- to 10-Hz frequency sweep with a constant 4% strain. Storage (G′) and loss (G″) moduli were reported (*n* = 3 per gel formulation). A one-way analysis of variance (ANOVA) was used to compare storage moduli.

### Nerve viability assay

Outgrowth from the spheroids in coculture was assessed using calcein acetoxymethyl ester from the LIVE/DEAD Viability Kit (Thermo Fisher Scientific, Waltham, MA). Constructs were incubated in culture media containing the live stain for 30 min and then imaged with the Nikon AZ100 (Tokyo, Japan) microscope at 0, 5, 10, 15, and 20 days in vitro (DIV) for dumbbell constructs and 0, 10, and 20 DIV for L-shaped constructs. The same construct was imaged at each time point to assess changes in outgrowth over time. Data from DIV 10 are shown in fig S1.

### Neurite outgrowth tracing

The directionality of tissue growth was characterized by tracking the live migration of fluorescent neurites emanating from DRG and SCDH tissues over time. AAV9-Syn-GFP viral particles (Vigene Biosciences, CV17001-AV9; Rockville, MD, USA) were incorporated during spheroid formation with a multiplicity of infection (MOI) of 2 million. The plasmid carried by this virus results in expression of GFP under control of the neuron-specific synapsin I promoter. Infected spheroids were then washed in excess growth media and seeded in dual-hydrogel constructs. GFP-expressing DRG and SCDH spheroids were seeded both alone (monoculture) and in coculture with an uninfected spheroid of the other tissue type. Three replicate cultures of each of the four conditions were analyzed including DRG(GFP) monocultures, SCDH(GFP) monocultures, DRG(GFP)-SCDH cocultures, and DRG-SCDH(GFP) cocultures. After 17 days of microphysiological nerve growth, GFP expression was imaged live on a fluorescent microscope (×2 magnification with ×5 optical zoom). The resulting images were then thresholded in ImageJ before quantification. All images were first processed by standard contrast limited adaptive histogram equalization to reduce blur, and a region of interest (ROI) was created to isolate the nerve growth channel. The auto-threshold function was applied to this ROI across all monoculture images to obtain an unbiased estimation of the appropriate threshold value. The estimated threshold value was averaged across all three DRG monocultures and separately averaged across all three SCDH monocultures. The average DRG monoculture threshold was then applied to all GFP-expressing DRG monocultures and cocultures, and the average SCDH monoculture threshold value was applied to all GFP-expressing SCDH monocultures and cultures. A binary mask was then created from the thresholded image to assign each pixel as either supra- or subthreshold. Last, ROIs corresponding to sequential 0.8-mm bins spanning the entire construct were created, and suprathreshold pixels were totaled in each bin to obtain a total above-threshold pixel count at discrete, increasing distances from GFP-expressing spheroids.

### Microphysiological nerve morphology

A batch of DRG and SCDH spheroids were generated, and 20% of the spheroids were infected with AAV9-Syn-GFP with an MOI of 2 million to reduce the proportion of infected neurons and enable visualization of individual neurons. Spheroids were then washed and cocultured in dual-hydrogel constructs for 17 days. Live constructs were then imaged with three-dimensional confocal microscopy on a Nikon confocal microscope to analyze both cellular and tissue-level morphology. For gross tissue morphology, three adjacent *z* stacks encompassing the entire vertical growth of the culture were imaged at ×10 magnification, stitched together, three-dimensionally rendered with alpha blending, and depth coded in Nikon Elements software to produce a three-dimensional map of culture thickness. *Z* stacks encompassing the DRG and SCDH were also separately imaged at ×20 for morphological analysis of individual neurons. Maximum intensity projections through select 20-μm vertical sections of the *z* stacks were generated to highlight individual neuronal soma and proximal neurite projections. To calculate average somal size, the diameter of all soma with distinct borders was measured throughout the *z* stack encompassing three DRG and SCDH spheroid regions from three independent cultures. The average somal diameter was calculated for each spheroid resulting in an “*n*” of 3 for statistical analysis.

### Preparation and imaging of whole-construct immunofluorescent analysis

Mature constructs were removed from the incubator and washed three times for 5 min with PBS to remove growth media from the dual-hydrogel scaffolds. Constructs were then fixed with ice-cold 4% paraformaldehyde for 20 min and washed three times for 10 min with ice-cold PBS to remove excess fixative. After immunostaining, cultures were mounted in ProLong Glass antifade mounting media with NucBlue DNA stain on a microscope slide and covered with a coverslip. Three-dimensional antibody staining was visualized on an Olympus confocal microscope. Three adjacent image stacks were generated at ×10 magnification and stitched together in cellSens to encompass the entire nerve construct. Images presented are maximum intensity projections through the resulting stitched image stack.

### Preparation and imaging of immunofluorescent staining of cryosections

Mature constructs were fixed with 4% paraformaldehyde for 30 min at 4°C. The solution was removed, and constructs were washed three times for 5 min with ice-cold PBS to remove excess fixative. Constructs were then sequentially submerged for 24 hours in 15 and 30% sucrose solution in PBS at 4°C. Sucrose solution was removed, and wells were filled with optimal cutting temperature compound (Thermo Fisher Scientific) and placed in a dry ice/isopentane bath for 5 min. After freezing the construct, they were stored at −80°C until cryosectioning. Constructs were cryosectioned perpendicular to the axis of neurite growth, creating 10- to 20-μm-thick slices, which were transferred onto positively charged microscope slides. Sides were stored at −30°C until immunostaining. After immunostaining, sections were mounted in ProLong Glass antifade mounting media with NucBlue DNA stain on a microscope slide and covered with a coverslip. Sections were imaged in cellSens software on an Olympus confocal microscope at ×40 magnification and ×3 digital zoom.

Images were then thresholded in ImageJ for colocalization analysis. Images representing each stain in the SCDH were individually opened, and the threshold was increased to drop out faint background staining. Images were then converted to a binary mask (1, above; 0, below threshold), and the total number of above-threshold pixels in the image was counted to quantify total staining for each antibody. Binary images representing individual channels were then multiplied together in a pixelwise fashion to generate a binary colocalization map (1, colocalization; 0, no colocalization). The total number of colocalized pixels was divided by the total number of above-threshold pixels in the individual channel images to calculate percent colocalization. To ensure consistency in the distribution of staining intensities included in the analysis, the thresholds identified in SCDH images were then applied to the analogous images obtained from DRG spheroid cryosections and the colocalization analysis was repeated.

### Immunofluorescent staining of whole constructs and cryosections

Tissue was blocked and permeabilized in 5% goat serum and 0.3% Triton X-100 in PBS for 1 hour, incubated with primary antibodies overnight in 5% goat serum and 0.3% Triton X-100 in PBS at 4°C, washed three times for 10 min with 0.3% Triton X-100 in PBS, incubated with secondary antibodies overnight in 5% goat serum in PBS at 4°C, and washed three times for 10 min in PBS. All steps were performed at room temperature with gentle agitation unless otherwise noted. Primary antibodies and dilutions included mouse anti–β-III-tubulin at 1:1000 (Abcam, ab78078), chicken anti-MAP2 at 1:5000 (Novus Biologicals, NB300-213), rabbit anti-peripherin at 1:1000 (Abcam, ab1530), rabbit anti vesicular GABA transporter at 1:1000 (Novus Biologicals, NBP2-20857), rabbit anti–synapsin I at 1:500 (Abcam, ab64581), mouse anti-PSD95 (NeuroMab, K28/43), and mouse anti CGRP at 1:500 (Abcam, ab81887). Secondary antibodies included Alexa Fluor 594 goat anti-mouse at 1:1000 (Abcam, ab150116), Alexa Fluor 488 goat anti-rabbit at 1:1000 (Abcam, ab150077), and Alexa Fluor 647 goat anti-chicken at 1:1000 (Abcam, ab150171).

### Optogenetic methods

AAV1-hSyn-NES-his-CaMPARI2-WPRE-SV40 was a gift from E. Schreiter (Addgene viral prep #101060-AAV1; http://n2t.net/addgene:101060; RRID:Addgene_101060), and AAV8-Syn-CHR2(H134R)-GFP was a gift from E. Boyden (Addgene viral prep #58880-AAV8; http://n2t.net/addgene:58880; RRID:Addgene_5880). Viral particles of both types were added during spheroid formation with an MOI of 200,000. Cultures were seeded and matured for 3 weeks. Baseline red fluorescence was imaged with both 3- and 10-s exposures before photostimulation using a Nikon AZ100 fluorescent microscope (×2 magnification with ×5 or ×8 digital zoom). All constructs across all experiments received an identical photostimulation protocol, during which the entire construct was illuminated for 3 s with 488-nm light focused through the fluorescent microscope (×2 magnification and ×5 digital zoom) to activate the CHR2-GFP fusion protein and 405-nm light was simultaneously applied with a 50-mW laser-emitting diode focused on the spheroid ROI to provide CaMPARI-2 photoconversion light. Post-photostimulation red fluorescence was again imaged with methods identical to baseline imaging. The integrated pixel intensity for an ROI encompassing each spheroid of interest was quantified in Fiji (ImageJ), and post-photostimulation intensity was normalized to prestimulation intensity to obtain a final value for fold change in integrated pixel intensity.

### Microphysiological field potential recording

For all electrophysiological experiments, mature constructs were submerged in room-temperature artificial cerebrospinal fluid (aCSF) bath composed of 170 mM NaCl, 7 mM KCl, 37 mM NaHCO_3_, 0.91 mM Na_2_HPO_4_**·**7H_2_O, 14 mM d-glucose, 4 mM MgSO_4_, and 2 mM CaCl_2_ in deionized water and continuously bubbled with 95% O_2_ and 5% CO_2_. A concentric bipolar stimulating electrode (FHC Inc., Bowdoin, ME, USA) was inserted into the nerve tissue at the desired stimulation site. The stimulus-response protocol was executed with LabChart Software (AD Instruments, Colorado Springs, CO, USA) including voltage steps with 0- to 10-V, 15-V, 20-V, 30-V, and 40-V amplitudes. Resulting field potentials were recorded by a platinum wire (A-M Systems, Sequim, WA), inserted into a pulled-glass micropipette filled with aCSF with resistance adjusted to 1 to 2 megohms. The tip of the glass recording pipette was inserted into nerve tissue at the desired recording site. Bioelectric signals were amplified with a Model 3000 AC/DC Differential Amplifier (A-M Systems, Sequim, WA) set at 100× gain and 0.1-Hz high-pass and 3-kHz low-pass filtering; electrical interference was removed with the Hum Bug Noise Eliminator (Quest Scientific, North Vancouver, Canada), and traces were digitized with the PowerLab analog-to-digital converter (AD Instruments), recorded in LabChart, and exported to Igor Pro (version 8; WaveMetrics Inc., Lake Oswego, OR, USA) for quantitative analysis. The 10 replicate traces obtained at each voltage step were averaged into a single trace representative of each step for analysis.

Field potential production was initially characterized in monocultures. DRG and SCDH monocultures were stimulated in the nerve growth region (concentrated nerve fibers) while recording in the spheroid region (concentrated neuronal soma) and stimulated in the spheroid region while recording in the nerve growth region (fig. S3, A, B, D, and E). When recording in cocultures, one spheroid was electrically stimulated, while resulting field potentials were recorded in the adjacent spheroid region (fig. S3, C, F, and G). Additional constructs were generated that permitted bidirectional growth of DRG but not SCDH spheroid tissue, and the waveform produced in the SCDH after short-, medium-, and long-distance stimulations was recorded and compared to infer the potential contribution of direct electrical activation of SCDH tissue to the DRG-SCDH synaptic waveform (fig. S3, H and I).

### Synaptic fatigue

In DRG-SCDH cocultures, 20-V, 200-μs square-wave stimulations were delivered to DRG tissue sequentially at 10, 20, and 25 Hz for 1 s while recording waveform production in the SCDH. The amplitude of each waveform component (N1, N2, and P1) was measured in Igor Pro for each response to the first 10 successive stimulations at each frequency. The amplitude of each component at each successive stimulation was then subtracted from and normalized to its baseline amplitude (after the first stimulation in that train) to calculate the percent change from baseline of each peak after each stimulation in each frequency train.

### Pharmacological challenges

DRG-SCDH cocultures were further challenged through specific pharmacological inhibition of glutamatergic and GABAergic neurotransmission to confirm the synaptic nature of the DRG-SCDH waveform. Constructs were removed from culture media and equilibrated in aCSF for 30 min, and a baseline stimulus-response curve was performed. Cultures were then perfused for 10 min with aCSF containing various pharmacological treatments, and the stimulus-response curve was repeated and compared to the baseline data to isolate drug-dependent effects. aCSF composed of 1 mM EDTA (MilliporeSigma) and 0 mM CaCl_2_ was perfused to inhibit all synaptic transmission. aCSF containing 10 μM CNQX disodium salt hydrate (MilliporeSigma) was perfused to inhibit AMPA/kainate receptor–dependent glutamatergic neurotransmission. (+)-Bicuculline (100 μM; MilliporeSigma) was perfused to inhibit GABA_A_R-dependent GABAergic neurotransmission. Tetrodotoxin (1 μM; Abcam) was perfused to inhibit all voltage-gated sodium channel–dependent CAP conduction. CNQX, bicuculline, and tetrodotoxin were dissolved in DMSO at 1000× and diluted 1:1000 in aCSF when used. DMSO concentrations were maintained at 0.1% throughout all pharmacological experiments.

The waveform corresponding to each voltage step under baseline and drug-treated conditions was quantitatively analyzed with a custom algorithm written in Igor Pro that identifies the location of the N1 peak, the N2 peak, and the P1 peak. Accurate peak selection was confirmed by a scorer blind to the treatment groups. The maximum amplitude of each peak, the latency to each peak, the difference in amplitudes between each peak, the difference in latencies between each peak, the integrated AUC between N2 and P1 (P1-early), the integrated AUC between P1 and the end of the recording (P1-late), and the integrated area from N2 to the end of the trace (P1-total) were automatically calculated for each trace. There was no indication of changes in presynaptic sensitivity following treatment with EDTA, CNQX, or bicuculline; therefore, maximal (40 V) stimulation traces were directly compared between baseline and drug-treated stimulus response curves.

### Comparison of analgesics

Three commonly used analgesic compounds of different classes were applied to DRG-SCDH cocultures to characterize their effects on microphysiological afferent pain signaling. A local anesthetic, (lidocaine), an α_2_ agonist (clonidine), and the prototypical opioid analgesic (morphine) were perfused in aCSF at increasing concentration to identify the lowest effective dose. DRG-SCDH constructs were again equilibrated in aCSF for 30 min to achieve a stable baseline. Lidocaine (MilliporeSigma) was first dissolved in DMSO and diluted into aCSF to a final concentration of 10 μM, 100 μM, 500 μM, and 1 mM. Clonidine (MilliporeSigma) was dissolved directly in aCSF (with 0.1% DMSO) at 100 μM and diluted to concentrations of 100 nM, 1 μM, 10 μM, and 100 μM. Morphine sulfate (provided by the NIH National Institute on Drug Abuse) was dissolved directly in aCSF (with 0.1% DMSO) and diluted to concentrations of 1, 10, 100, and 500 μM. A stimulus-response curve was performed after application of each dose of each drug and compared to baseline recordings to isolate drug-dependent effects (fig. S5). A washout was then performed to ensure the reversibility of the drug-dependent effects (fig. S6).

The lowest effective doses for each drug were then replicated four times for in-depth quantification and statistical analysis. Constructs were placed on the rig and equilibrated in aCSF for 30 min, and a stimulus-response curve was obtained at baseline. A sham treatment was then performed in which the entire recording bath was drained and replaced with aCSF containing vehicle (0.1% DMSO), the construct was incubated for 10 min, and a second stimulus-response curve was obtained. The entire bath was drained again and was replaced with aCSF containing the analgesic treatment; then, the construct was incubated for 10 min, and a third stimulus-response curve was obtained.

The waveform corresponding to each voltage step under baseline, sham, and analgesic-treated conditions was quantitatively analyzed with a custom algorithm written in Igor Pro that identifies the location of the N1 peak, the N2 peak, and the P1 peak. Accurate peak selection was confirmed by a scorer blind to the treatment groups. The maximum amplitude of each peak, the latency to each peak, the difference in amplitudes between each peak, the difference in latencies between each peak, the integrated AUC between N2 and P1 (P1-early), the integrated AUC between P1 and the end of the recording (P1-late), and the integrated area from N2 to the end of the trace (P1-total) were automatically calculated for each trace.

After initial analysis, it was apparent that analgesic treatments affected the presynaptic fiber volley peak (N1) in addition to the synaptic components N2 and P1. A second analysis was performed to control for apparent analgesic-induced differences in afferent input sensitivity. The waveform produced after 40-V stimulation of analgesic-treated constructs was matched with the lower-intensity sham waveform with the most similar N1 amplitude. This comparison aligns traces with the most similar apparent strength of afferent input, and any remaining differences are more likely direct effects on synaptic transmission. Last, analgesic-induced differences in the sensitivity of the three different waveform components were directly calculated. The maximal amplitude of N1, N2, and P1 was obtained from the 40-V analgesic-treated traces. The sham-treated traces that resulted in the most similar amplitudes for each of these components were then identified.

### Statistical analysis

The GFP-expressing neurite tracing experiment was designed to separately compare neurite outgrowth from DRG and SCDH monocultures to growth of these same tissues in DRG-SCDH coculture. Two two-way mixed model ANOVAs with the between-subjects factor of “culture type” (levels include “monoculture” and “coculture”) and the within-subjects factor of “location” (levels include bins of increasing distances from GFP-expressing spheroid in 0.8-mm increments) were used to identify significant differences in GFP-expressing neurite density over increasing distance from GFP-expressing DRG and SCDH spheroid centers. Following identification of significant culture type by location interaction, least significant difference post hoc tests were performed to identify significant differences between levels of culture type at each level of location.

A significant difference in the average somal size between DRG and SCDH neurons was tested with a Student’s *t* test. The diameters of all clearly distinguishable neuronal soma were measured across three different DRG and SCDH spheroids. The average diameter of all soma within each construct was calculated, and these six average (three DRG and three SCDH) values were compared with a Student’s *t* test.

The optogenetic experiments were conducted with between-samples designs. The fold change in red fluorescence before and after photostimulation was compared between control cultures and groups of cultures that received different independent treatments. Thus, the difference in fold change between control and treatment groups was compared with an unpaired *t* test in four separate experiments. First, three DRG(CaMPARI-2) cultures were compared to three DRG(CHR2-GFP+CaMPARI-2) cultures. Images were collected with a 3-s exposure and ×2 magnification with ×8 zoom (Fig. 4, A and B). Second, eight identical DRG(CaMPARI-2) constructs were split into two groups, which were treated for 5 min with either 10 μM capsaicin or vehicle alone (0.1% DMSO) before photostimulation to record capsaicin-induced calcium currents. Images were collected with a 10-s exposure at ×2 magnification with ×8 zoom (Fig. 4, A and C). Third, three DRG(WT)-SCDH(CaMPARI-2) were compared to three DRG(CHR2-GFP)-SCDH(CaMPARI-2). Images were collected with a 3-s exposure at ×2 magnification with ×5 zoom (Fig. 4, D and E). Last, eight identical DRG(CHR2-GFP)-SCDH(CaMPARI-2) cultures were split into two groups, which were treated with either 200 μM CNQX or vehicle alone (0.28% DMSO) for 15 min before photostimulation to inhibit glutamatergic neurotransmission. Images were collected with a 3-s exposure and ×2 magnification with ×8 zoom (Fig. 4, F and G).

Electrophysiological experiments were conducted according to within-samples designs. Each described metric was measured before and after treatment from electrophysiological waveforms derived from the same construct. To account for between-construct differences in the absolute magnitude of the waveform (independent of treatment), the value of the metric after treatment was subtracted from its value before treatment to obtain a “change in metric” value. The change in each metric was then compared with a one-sample *t* test to a value of zero, which would be indicative of no change. The synaptic fatigue experiment was replicated in three independent cultures. The percent change from baseline was averaged across experiments, and significant changes in each described metric were identified with a one-sample *t* test versus 0% (no change) after the 10th stimulation (Fig. 6B). Pharmacological inhibition of neurotransmission was replicated four or five times. Four independent constructs were treated with EDTA. Five constructs were treated with CNQX, and four constructs were treated with bicuculline. The percent change from baseline of each described metric was averaged across constructs, and significant changes were identified with a one-sample *t* test versus 0% (Fig. 7, D to F). Each analgesic was applied to four independent cultures. The change in each of these values between baseline and sham treatments and between sham and analgesic treatments at each stimulation intensity was averaged across constructs, and a one-sample *t* test versus 0 (no change) was performed to identify significant sham treatment– or analgesic treatment–induced changes at each voltage (fig. S7). To control for apparent differences in presynaptic desensitization, differences in each described metric between the 40-V analgesic-treated waveform and the N1-matched sham waveform were averaged across constructs and significant differences were determined with a one-sample *t* test versus 0 (Fig. 8, D to F). Last, the percent change in stimulus intensity required to evoke this response of similar amplitude was averaged across constructs for each waveform component, and statistical significance was determined with a one-sample *t* test versus 0% (Fig. 8G).

## SUPPLEMENTARY MATERIALS

Supplementary material for this article is available at http://advances.sciencemag.org/cgi/content/full/7/35/eabj2899/DC1
